# Snake scales, partial exposure, and the Snake Detection Theory: A human event-related potentials study

**DOI:** 10.1038/srep46331

**Published:** 2017-04-07

**Authors:** Jan W. Van Strien, Lynne A. Isbell

**Affiliations:** 1 Department of Psychology, Education, and Child Studies, Erasmus University Rotterdam, The Netherlands; 2 Department of Anthropology and Animal Behavior Graduate Group, University of California, Davis, USA

## Abstract

Studies of event-related potentials in humans have established larger early posterior negativity (EPN) in response to pictures depicting snakes than to pictures depicting other creatures. Ethological research has recently shown that macaques and wild vervet monkeys respond strongly to partially exposed snake models and scale patterns on the snake skin. Here, we examined whether snake skin patterns and partially exposed snakes elicit a larger EPN in humans. In Task 1, we employed pictures with close-ups of snake skins, lizard skins, and bird plumage. In task 2, we employed pictures of partially exposed snakes, lizards, and birds. Participants watched a random rapid serial visual presentation of these pictures. The EPN was scored as the mean activity (225–300 ms after picture onset) at occipital and parieto-occipital electrodes. Consistent with previous studies, and with the Snake Detection Theory, the EPN was significantly larger for snake skin pictures than for lizard skin and bird plumage pictures, and for lizard skin pictures than for bird plumage pictures. Likewise, the EPN was larger for partially exposed snakes than for partially exposed lizards and birds. The results suggest that the EPN snake effect is partly driven by snake skin scale patterns which are otherwise rare in nature.

Both humans and other primates can detect snakes faster than other, less life-threatening stimuli^1^,^2^. The “Snake Detection Theory”^3^,^4^ argues that snakes were ultimately responsible for the origin of primates by acting as a selective pressure in the modification and expansion of primate visual systems such that vision is now their predominant sensory interface with the environment. Predatory pressure from snakes is proposed to have contributed to primate visual modification and expansion by weeding out those individuals with poorer ability, and favoring those with better ability, to visually detect motionless snakes.

Electrophysiological evidence in support of greater visual sensitivity to snakes than to other stimuli is growing both in humans and other primates. Le and colleagues^5^ measured neuronal responses in the medial and dorsolateral pulvinar of macaques (*Macaca fuscata*) that likely had no exposure to snakes before the experiment. Their study revealed the existence of pulvinar neurons that responded selectively faster and stronger to images of snakes than to images of angry and neutral monkey faces, monkey hands, and simple geometrical shapes. Neurons in the pulvinar were also found to respond more strongly (but not more quickly) to images of snakes in striking postures than in resting postures^6^. As the authors noted, the pulvinar is part of a fast visual information processing pathway also involving the retina and superior colliculus, allowing for rapid, automatic visual detection of fear-related stimuli^7^,^8^. In a third study, Le and colleagues^9^ demonstrated that images of snakes elicited gamma (30–80 Hz) oscillations in macaque pulvinar neurons in an early time window (0–200 ms after stimulus onset), whereas monkey faces elicited gamma oscillation in a later time window (300–500 ms after stimulus onset). They interpret the early gamma oscillations for snakes as indicative of feedforward processes that facilitate processing in the visual cortex.

Recent studies using event-related potentials (ERPs) have also tested predictions derived from the Snake Detection Theory in humans^10^,^11^,^12^. In the first study, participants watched the random rapid serial visual presentation (RSVP^13^) of 600 snake pictures, 600 spider pictures, and 600 bird pictures at a rate of 3 pictures per second^10^. An ERP component peaking around 225–300 ms after stimulus onset, the so-called Early Posterior Negativity (EPN), was measured at lateral occipital sites. The EPN amplitude was largest for snake pictures, intermediate for spider pictures and smallest for bird pictures. The EPN reflects the early selective visual processing of emotionally significant information, a process that is not altered by habituation^14^. It represents the selection of stimuli for further processing^15^ and is associated with the functioning of the basic motivational systems of approach and avoidance. The EPN is augmented particularly by stimuli of evolutionary significance^16^.

A second ERP study with the RSVP paradigm examined (a) whether the preferential activity in early visual processing is specific to snakes or is a categorical reptile effect and (b) whether disgusting animals such as slugs enhance early visual processing as well^11^. The EPN was significantly larger for snake pictures than for pictures of other reptiles and pictures of slugs, suggesting a snake-specific attention effect.

A third study examined to what extent the typical curvilinear body shape of snakes causes the enlarged EPN by employing the RSVP for pictures of threatening and nonthreatening species with or without typical body curvature (i.e., snakes, worms, spiders, and beetles)^12^. The EPN was significantly larger for snake pictures than for spider, worm, and beetle pictures, and for spider and worm pictures than for beetle pictures. The results suggest that curvilinear body shapes may partly drive the enhanced EPN. However, similarly shaped worms did not elicit a similar response, which suggests that the typical EPN response to snakes is not fully explained by their body shape.

In none of these studies was an association found between EPN amplitudes and participants’ reported fear for snakes, which supports the view that the EPN reflects the automatic first-stage visual processing of emotional cues, and this processing may be independent of consciously reported fear.

Finally, another RSVP study conducted in a different lab demonstrated larger EPN amplitudes to brightness-controlled grayscale pictures of snakes compared to spiders^17^. Taken together, the results of the above electrophysiological studies suggest that snake stimuli modulate the early capture of visual attention and that this attention is automatic and independent of consciously reported fear.

Behavioral research on the visual sensitivity of primates to snakes is consistent with electrophysiological studies. When captive rhesus macaques (*M. mulatta*) were exposed to a snake model in a striking posture, they responded more strongly than when they were exposed to snake models in coiled or extended sinusoidal postures^18^. Interestingly, a partially exposed snake model elicited responses in these animals that were comparable to their responses to the striking snake model. In nature, snakes are often difficult to see because their color patterns often blend in with the surrounding vegetation and they can position themselves within the vegetation so that their bodies are only partially visible. Although snakes are characterized by having a long, curvilinear, legless, body shape, under such conditions, body shape would be unreliable as a visual cue. However, smaller visual stimuli such as scale patterns might be highly reliable. Not only do snakes have unique scale shapes, these patterns are sufficiently small as to be visible even when snakes are partially exposed. A recent field experiment demonstrated that non-human primates are indeed highly responsive to partially exposed snakes. Wild vervet monkeys (*Chlorocebus pygerythrus*) were able to detect only 2.5 cm of snake skin in less than one minute^19^. Thus, snake scales alone (i.e., without curvilinear cues) were sufficient for detection. Further support for the role of scales in driving snake detection comes from research that has demonstrated that white-faced capuchin monkeys (*Cebus capucinus*) respond more strongly to snake models with scales than to white snake models without scales^20^. Here we examined whether humans are also highly responsive to partially exposed snakes and specifically to snake skin scales by employing EEG.

In Task 1, we used the RSVP of pictures with close-ups of snake skins, lizard skins, and bird plumages to examine the influence of snake-scale patterns on the human EPN. In Task 2, we employed the RSVP of pictures with partially exposed snakes, lizards, and birds to examine whether responses to pictures of partially exposed animals are similar to those obtained in previous research with fully exposed animals. Based on earlier EEG research and the observations with non-human primates, we expected larger (more negative) EPN amplitudes to pictures of snake skins compared to lizard skins and bird feathers and we expected larger EPN amplitudes in response to partially exposed snakes than to partially exposed lizards and birds. In addition, participants filled out fear questionnaires, and rated the valence and arousal of the individual pictures for these species. This was done to determine whether conscious emotional perceptions of snakes are associated with the EPN amplitudes. Based on previous research, we expected no correlations between conscious emotional perceptions of snakes and the EPN responses to them.

## Method

### Participants

Twenty-four university students (12 men, 12 women) participated in both tasks for course credits or a monetary reward. Ages ranged from 18 to 25 years, with a mean age of 19.67 years. The study was approved by the Institute of Psychology ethics committee of the Erasmus University Rotterdam and conducted in accordance with the regulations of the Declaration of Helsinki. All participants provided written informed consent.

### Tasks and questionnaires

**Figure 1:**
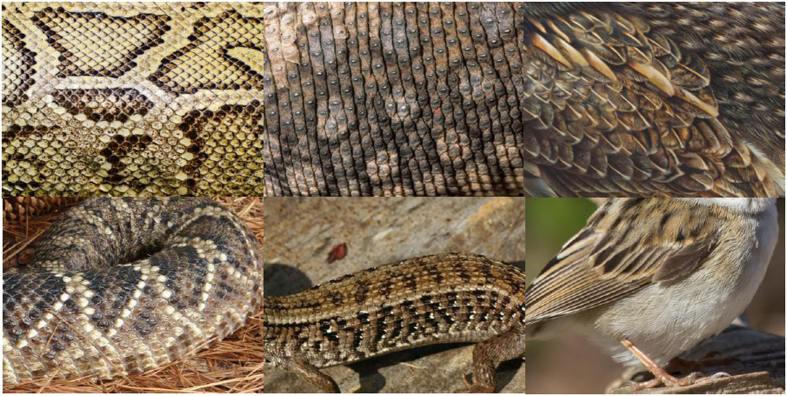
Illustrative examples of stimuli depicting snake skin, lizard skin, and bird plumage stimuli (top row) and examples of stimuli depicting partially exposed snakes, lizards, and birds (bottom row). For copyright reasons, the depicted photographs are public domain (pixabay.com); they are similar to the actual stimuli, but were not used in the experiment.

Participants were seated in a dimly lit room and were told to attentively watch the continuous RSVP of the various stimulus categories. In Task 1, the RSVP consisted of 300 pictures of snake skins, 300 pictures of lizard skins, and 300 pictures of bird feathers. In Task 2, the RSVP consisted of 300 pictures of partly exposed snakes, 300 pictures of partly exposed lizards, and 300 pictures of partly exposed birds. For each stimulus category, 10 different pictures were shown 30 times. Pictures were obtained from various Internet sites. In Task 1, each picture showed a close-up of a snake skin, a lizard skin, or the plumage of a bird. In Task 2, each picture showed a partly exposed snake, lizard, or bird body against a natural background. No animal heads were shown on these pictures. Examples of the stimulus categories are given in Fig. 1. The pictures were shown at a distance of 120 cm on a 20-inch PC monitor with a resolution of 1024 × 768 pixels. Pictures had a size of 600 × 450 pixels, and were displayed against a medium grey background. The presentation rate was 3 pictures per second. In each task, the pictures were presented randomly within each cycle of 30 unique pictures (30 cycles) without gaps until all pictures were presented. The order of Tasks 1 and 2 was fixed because we did not want to prime the viewing of the skins and plumages in advance by showing snake, lizard, and bird pictures. There was a short rest between the block of 900 picture presentations in Task 1 and the block of 900 picture presentations in Task 2.

Following the experimental run, participants completed a computerized Self-Assessment Manikin (SAM) questionnaire^21^ regarding valence and arousal ratings of all pictures on a 9-point scale. For these ratings, the order of pictures was random for each participant. After the SAM questionnaire, participants rated their fear of snakes, lizards and beetles on a 15-item paper and pencil questionnaire for each category. The questionnaires were adapted versions of the Spider Phobia Questionnaire (SPQ^22^,^23^). With the statements rated on a 4-point scale, scores on each questionnaire could range from 0 (no fear) to 45 (very high fear). Half of the participants completed the snake questionnaire first and the lizard questionnaire last, the other half completed the questionnaires in opposite order.

### EEG recording and analysis

EEG activity was recorded using a BioSemi Active-Two system from 32 pin type active Ag/AgCl electrodes mounted in an elastic cap. An active electrode (common mode sense) and a passive electrode (driven right leg) were used to comprise a feedback loop for amplifier reference. To record Electrooculogram (EOG) activity, active electrodes were placed above and beneath the left eye, and at the outer canthus of each eye. The EEG and EOG signals were digitized with a 512-Hz sampling rate, a low-pass filter of 134 Hz, and 24-bit A/D conversion.

Offline, the EEG signals were referenced to an average reference and phase-shift-free filtered with a band pass of 0.10–30 Hz (24 dB/Oct). Horizontal and vertical eye movements were corrected using the Gratton and Coles algorithm^24^. ERP epochs were extracted with a 380-ms duration and beginning 50 ms before stimulus onset. The ERP signals were defined relative to the mean of this 50-ms pre-stimulus baseline period. For each of the two tasks, average ERPs were computed for each participant and each condition (snake, lizard, bird). Epochs with a baseline-to-peak amplitude difference larger than 100 μV on any channel were omitted from averaging. The mean percentage of valid epochs at relevant electrodes was more than 99% for each stimulus category. The EPN was scored at posterior electrodes (O1, Oz, O2, PO3, PO4, P7, P8) and was measured, in accordance with previous research^10^,^11^,^12^,^25^,^26^, as the mean activity in the 225–300 ms time window after stimulus onset.

### Statistical analyses

**Figure 2:**
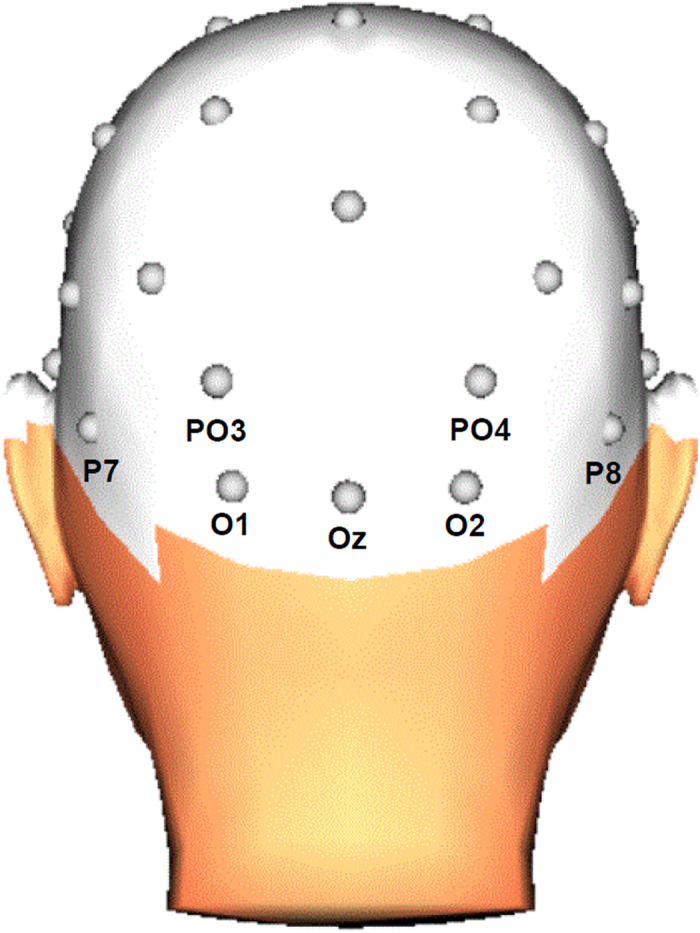
Diagram of the EEG electrodes included in the statistical analyses. 3D head view created with BrainVision Analyzer 2.1, Brain Products GmbH, Gilching, Germany.

For the valence and arousal ratings of the stimuli of each task, and for the fear measures, separate repeated-measures analyses of variance (ANOVAs) were employed with stimulus category (snake, lizard, bird) as one factor. For the EPN components, separate repeated-measures ANOVAs were conducted for each task, with stimulus category (snake, lizard, bird) and electrode (O1, Oz, O2, PO3, PO4, P7, P8) as factors (see Fig. 2 for the electrode positions). At the occipital and lateral occipito-parietal electrodes included in the analysis, the EPN is typically modulated by stimuli of evolutionary significance^11^. When appropriate, Greenhouse-Geisser correction was applied. To explore the relationship between reported fear for the three animal categories and the EPN amplitudes, we calculated the rank correlations (Spearman’s rho) between questionnaire scores and EPN amplitudes for snake skins, lizard skins, and bird feathers, and for partially exposed snakes, lizards, and birds, respectively. To reduce the total number of correlations, we employed one occipital cluster (comprising the seven included electrodes) for the EPN amplitude measure.

## Results

### Fear measures

Scores on the snake questionnaire ranged from 3 to 31, with a mean of 13.0 (SD = 6.3). Scores on the lizard questionnaire ranged from 1 to 19, with a mean of 9.5 (SD = 4.1). On the bird questionnaire the scores ranged from 0 to 25, with a mean of 6.0 (SD = 5.3). We found a significant stimulus category effect, F(2, 46) = 16.06, epsilon = 0.674, p < 0.001. Bonferroni corrected comparisons showed that participants had more fear of either snakes or lizards than of birds (both p-values < 0.001). Fear of snakes was larger than fear of lizards (p = 0.031).

### Valence and arousal ratings

The mean valence and arousal ratings for the different stimulus categories in both tasks are given in Table 1. For the stimuli of Task 1, the category effects were significant for both valence, F(2,46) = 7.14, epsilon = 0.775, p = 0.005, and arousal, F(2,46) = 4.98, epsilon = 0.662, p = 0.024. Bonferroni-corrected pairwise comparisons revealed that pictures of bird feathers tended to be rated as more pleasant (p = 0.084) and less arousing (p = 0.094) than pictures of snake skins and were rated as more pleasant (p = 0.003) and less arousing (p = 0.035) than pictures of lizard skins. There were no differences in valence and arousal ratings for pictures of snake and lizard skins. For the stimuli of Task 2, the category effects were also significant for both valence F(2,46) = 15.20, epsilon = 0.781, p < 0.001, and arousal, F(2,46) = 7.85, epsilon = 0.722, p = 0.004. Bonferroni-corrected pairwise comparisons revealed that bird pictures were rated as more pleasant (both p-values ≤ 0.001) and less arousing (both p-values ≤ 0.025) than snake and lizard pictures. There were no differences in valence and arousal ratings for snake and lizard pictures.

### EPN Task 1

**Figure 3:**
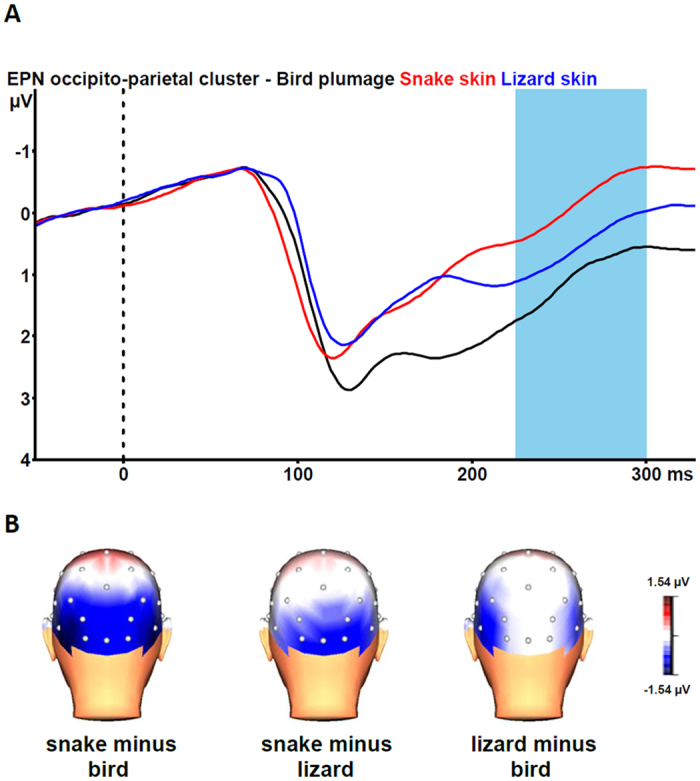
(**A**) Grand-average waveforms for the early posterior negativity (EPN) in response to pictures of snake skin (red line), lizard skin (blue line), and bird plumage (black line) at the occipito-parietal cluster (collapsed across O1, Oz, O2, PO3, PO4, P7, P8). The vertical bar depicts the EPN time window. (**B**) Grand-average topographic maps of the differences in the 225–300 ms mean area amplitudes between pictures of snake skin vs. bird plumage (left), snake skin vs. lizard skin (middle), and lizard skin vs. bird plumage (right). 3D head view created with BrainVision Analyzer 2.1, Brain Products GmbH, Gilching, Germany.

Figure 3A shows the grand average EPN potentials at the occipital cluster (O1, Oz, O2, PO3, PO4, P7, P8) for pictures of snake skin, lizard skin, and bird plumage. Snake skin pictures yielded the largest negative-going wave form, lizard skin pictures an intermediate negative-going wave form, and plumage pictures the smallest negative-going wave form. The ANOVA revealed a significant stimulus category effect, F(2, 46) = 35.84, epsilon = 0.912, *p* < 0.001. Bonferroni-corrected pairwise comparisons revealed that the EPN was significantly more negative for snake skin pictures than for lizard skin pictures and bird plumage pictures (both p-values < 0.001). Lizard skin pictures evoked a more negative EPN than plumage pictures (p = 0.013).

The interaction of stimulus category and electrode was also significant, F(12, 276) = 5.13, epsilon = 0.432, p < 0.001. As can be seen from Fig. 3B, the stimulus category effects are most pronounced for the snake versus bird and the snake versus lizard contrasts at all occipito-parietal electrodes. The lizard versus bird contrast is most pronounced at parietal (P7, P8) electrodes. Subsequent single-electrode analyses revealed significant stimulus category effects at all electrodes (all *p*-values ≤ 0.003). Bonferroni-corrected pairwise comparisons revealed that snake pictures elicited larger EPN amplitudes than lizard and bird pictures at all electrodes (all *p*-values ≤ 0.038). Lizard pictures elicited larger EPN amplitudes than bird pictures at O1, PO3, P7, and P8 (all p-values ≤ 0.013). At Oz, O2, and PO4, no differences between EPN amplitudes to lizard and bird pictures were found (all *p*-values ≥ 0.359).

### EPN Task 2

**Figure 4:**
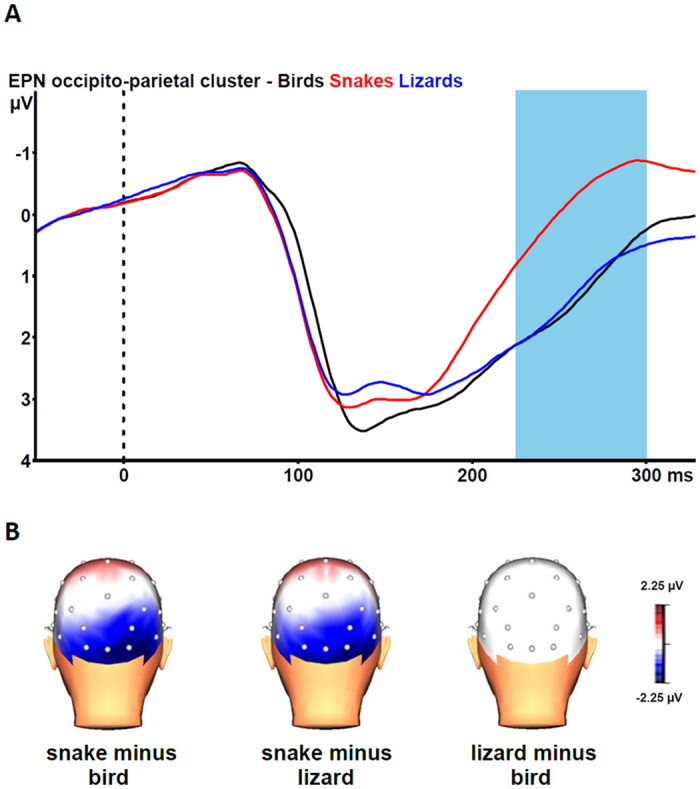
(**A**) Grand-average waveforms for the early posterior negativity (EPN) in response to pictures of partly exposed snakes (red line), lizards (blue line), and birds (black line) at the occipito-parietal cluster (collapsed across O1, Oz, O2, PO3, PO4, P7, P8). The vertical bar depicts the EPN time window. (**B**) Grand-average topographic maps of the differences in the 225–300 ms mean area amplitudes between pictures of partly exposed snakes vs. birds (left), snakes vs. lizards (middle), and lizards vs. birds (right). 3D head view created with BrainVision Analyzer 2.1, Brain Products GmbH, Gilching, Germany.

Figure 4A shows the grand average EPN potentials at the occipital cluster for pictures of partly exposed snakes, lizards, and birds. Snake pictures yielded the most negative-going wave form, compared the other two picture categories. The ANOVA revealed a significant stimulus category effect, F(2, 46) = 42.36, epsilon = 0.840, p < 0.001. Bonferroni-corrected pairwise comparisons revealed that the EPN was significantly more negative for snake pictures than for the two other categories (all p-values < 0.001). For lizard and bird pictures, no difference in EPN amplitude emerged (p* *=* *1.000).

The interaction of stimulus category and electrode was also significant, F(10, 276) = 6.75, epsilon = 0.425, p < 0.001. As can be seen from Fig. 4B, the stimulus category effects are only observable for the snake versus lizard and the snake versus bird contrasts at the occipito-parietal sites. Subsequent single-electrode analyses revealed significant stimulus category effects at all electrodes (all p-values < 0.001). Bonferroni-corrected pairwise comparisons revealed that snake pictures elicited larger EPN amplitudes than lizard and bird pictures at all seven electrodes (all p-values ≤ 0.003). None of the electrodes showed a difference between EPN amplitudes to lizard and bird pictures (all p-values = 1.000).

### Correlation analyses

The rank correlations between the EPN cluster amplitude measures and the fear ratings for snakes, lizards, and birds are given in Table 2. Participants who reported greater fear of snakes exhibited larger (more negative) EPN amplitudes in response to pictures of snake skins. There was no association between snake fear and EPN amplitudes in response to partially exposed snakes. Participants who reported greater fear of lizard exhibited larger (more negative) EPN amplitudes in response to pictures of lizard skins and pictures of partially exposed lizard. There was no association between fear of birds and EPN amplitudes in response to pictures of bird plumage or pictures of partially exposed birds.

## Discussion

To examine the influence of snake-scale patterns on the human EPN, we employed the RSVP of pictures with close-ups of snake skins, lizard skins, and bird plumages (Task 1). We expected the largest EPN amplitudes in response to snake skins, as pictures of fully exposed snakes typically elicit more negative EPN amplitudes than pictures of other animals. To examine whether pictures of partially exposed animals resulted in the same snake effect that was obtained in previous research with fully exposed animals we further employed the RSVP of pictures with partially exposed snakes, lizards, and birds (Task 2).

Snake skin pictures elicited the largest EPN amplitudes, lizard skin pictures intermediate EPN amplitudes, and bird plumage pictures the smallest EPN amplitudes. This demonstrates a clear EPN snake effect for snake skin stimuli, which suggests that snake scales are capturing more automatic attention than lizard skins or bird plumage. We did not expect larger EPN amplitudes in response to lizard skin stimuli than to bird plumage stimuli, as lizards and birds are not typically threatening taxa for modern humans. Most probably, the close-ups of lizard skins somewhat resemble the snake skins, and thus trigger a heightened EPN amplitude. The effect for lizard skin versus bird plumage was observed at inferior lateral electrodes only (P7, P8), which suggests that a rather high level of visual processing is involved in the processing of lizard skin versus bird plumage. This activity at P7 and P8 probably involves the ventral route. The EPN effect for snake skin versus lizard skin and versus bird plumage was not only found at P7 and P8, but also at central and pericentral occipital electrodes (O1, Oz, O2, PO3, PO4). This may reflect a lower level of visual processing. Studies of non-human primates^5^,^9^ propose that at a subcortical level, at the pulvinar in particular, neurons may be specialized in detecting snake features, thus modulating subsequent early object visual processing^27^. Alternatively, or in addition, it could be that snake skin scales and patterns trigger specific neurons in the visual cortex that are sensitive to lines of different orientations or to diamond-shaped patterns^28^ thus allowing fast snake recognition.

The EPN results for the pictures with partially exposed animals clearly replicate previous findings with fully exposed animals^10^,^11^. Partially exposed snakes elicited larger EPN amplitudes compared to partially exposed lizards and birds. In contrast to Task 1, there were no differences in EPN responses to lizards and birds. These EPN results indicate that human attention is preferentially directed towards snakes compared to lizards and birds, even when snakes are only partially visible. This is in agreement with behavioral studies with captive rhesus macaques^18^ and wild vervet monkeys^19^ that showed that these animals responded strongly to a partially exposed snake model. As snakes are often hidden in vegetation, the detection of partially visible snakes in particular has high survival value. The absence of an enhanced EPN amplitude in response to partially exposed lizards suggests that when it is immediately obvious that the depicted animal is not a snake, the EPN will not increase. In Task 1 only the skin of a lizard was shown, whereas in Task 2 parts of the lizard’s legs were visible, which made the identification of the animal as a non-snake easier. These differences in ease of identification might also be reflected by the time course of the earlier ERP waveforms. From Fig. 1, it can be seen that the grand-average waveforms in response to snake and lizard skins on the one hand and bird plumage on the other hand start to diverge from about 120 ms onwards, with the difference between snake and lizard skins emerging only after about 200 ms. From Fig. 2, it can be seen that the grand-average ERP waveform in response to partially exposed snakes starts to diverge from ERP waveform in response to partially exposed lizards slightly earlier. Further research is needed to clarify the influence of discriminability between snakes and lizards on the time course of the early ERP components.

Previous EPN research^12^ has suggested that the typical curvilinear body shape of snake stimuli may partly drive the enhanced EPN in response to snake pictures. Compared to beetle pictures, the EPN was significantly larger for snake pictures and worm pictures, suggesting higher attentional capture by curvilinear creatures. Yet, the EPN in response to snake pictures was significantly larger than in response to worm pictures, indicating that snakes possess other threat-relevant cues than curvature alone. Here we found in Task 1 that the scale patterns of the snake skin suffice to boost the EPN response. Note that in Task 1 no curvature was visible. The present results suggest that scale patterns typical of snake skins are a more relevant threat-cue than the curvilinear body shape. This is in agreement with behavioral research with wild vervet monkeys^19^ which also demonstrated that snake scales alone were sufficient for snake detection. Indeed, because snakes are often hidden by vegetation, their scales are often the only visual cue available for their detection.

Self-reported fear was highest for snakes, intermediate for lizards, and lowest for birds. In general, the fear scores were rather low, which indicates that the large majority of our participants did not exhibit phobic-like fears for these animals. In addition, snake fear scores were associated with EPN amplitudes in response to pictures of snake skins, but not with EPN amplitudes in response to pictures of partially exposed snakes. Lizard fear scores were associated with EPN amplitudes in response to pictures of both lizard skins and partially exposed lizards. In previous studies significant correlations were not found between snake fear and EPN amplitudes^10^,^11^,^12^, but significant correlations were found between spider fear and EPN amplitudes^10^,^26^. The lack of an association between snake fear and EPN amplitudes may be a consequence of the fact that participants from northwestern European countries rarely or never engage snakes –as opposed to spiders– in the wild, and hence cannot exactly assess their actual fear of snakes. The lack of an association may indicate the innate nature of early attention to snakes. In the present study, the lack of an association between snake fear and the EPN amplitude in response to partially exposed snakes is in agreement with previous studies in which pictures of completely exposed snakes were shown^10^,^11^,^12^. It is not clear why an association is found when participants see only the skin of a snake. The significant correlations between lizard fear and the EPN amplitudes in response to both lizard skins and partially exposed lizards might be comparable to the significant associations found between spider fear and the EPN amplitude^10^,^26^. As lizards pose no real threat, early attention to lizards may be less innate and more dependent on conscious experience than early attention to snakes. There were no significant correlations between bird fear scores and EPN amplitudes in response to bird stimuli. This comes as no surprise, as most participants will not have any fear of birds, and birds do not appear to capture automatic attention.

Pictures of bird plumage and of partially exposed birds were rated as more pleasant and less arousing than snake and lizard stimuli. These valence and arousal ratings are comparable with the ratings in a previous study involving birds^10^, in which the highest (most pleasant) valence scores and lowest arousal scores were found for birds. The valence and arousal ratings did not differ for snake and lizard stimuli. In line with previous studies, this suggests that there is no systematic relationship of valence and arousal ratings with EPN amplitudes, which supports the notion that the EPN reflects the automatic first-stage processing of emotional cues^14^,^15^.

Here we used naturalistic stimuli (i.e., realistic pictures of snakes, lizards, and birds). It could be argued that low-level visual features such as color, luminance, and spatial frequency of the pictures might influence the EPN and should be controlled. We chose to employ ecologically valid stimuli instead, as low-level features as such may be inherent properties of the fear stimulus and important for threat detection. After all, the evolution of visual sensitivity in primates did not take place under controlled laboratory conditions. It will be worthwhile to examine the contribution of each of these low-level features to the EPN snake effect, but we expect that the effect of low-level features on the EPN in response to snake pictures will be marginal in most cases. Research in other laboratories reveals that the EPN in response to brightness-equated grayscale pictures^17^ and to luminance- and contrast-equated color pictures^25^ also yield a distinct EPN snake effect. In the present and previous research, Van Strien and colleagues have been using close-ups of snake skins, pictures of partially exposed snakes and pictures of fully exposed snakes and compared them to many other animal categories. All these pictures may have differed in low-level visual features, yet in all cases snakes elicit larger EPN amplitudes than other animal categories.

To conclude, by employing random RSVP of snake skin, lizard skin, and bird plumage pictures, we found that the EPN was significantly larger for snake skin pictures than for lizard skin and bird plumage pictures, and for lizard skin pictures than for bird plumage pictures. With the random RSVP of pictures of partially exposed snakes, lizards, and birds we found that the EPN was larger for partially exposed snakes than for partially exposed lizards or birds. While previous research has suggested that the snake’s curvilinear body shape may partially drive the boosted EPN response to snake pictures^12^, the present results, which are consistent with expectations of the Snake Detection Theory^3^,^4^, indicate that snake skin scales may be a more threat-relevant visual cue that is rapidly detected and attended to among humans.

## Additional Information

**How to cite this article**: Van Strien, J. W. and Isbell, L. A. Snake scales, partial exposure, and the Snake Detection Theory: A human event-related potentials study. *Sci. Rep.* **7**, 46331; doi: 10.1038/srep46331 (2017).

**Publisher's note:** Springer Nature remains neutral with regard to jurisdictional claims in published maps and institutional affiliations.

## Figures and Tables

**Table 1:** Participants’ mean valence and arousal ratings (and standard deviations) for the various stimulus categories.

Stimulus category	Valence (SD)	Arousal (SD)
Task 1: Skin/feathers		
snake	5.0 (1.4)	3.3 (2.0)
lizard	4.8 (1.0)	3.1 (1.6)
bird	5.6 (1.3)	2.6 (1.3)
Task 2: Partly exposed		
snake	4.4 (1.6)	4.3 (2.5)
lizard	4.6 (1.4)	3.7 (2.1)
bird	5.9 (1.5)	2.8 (1.9)

Valence and arousal ratings are based on a rating scale from 1 to 9.

**Table 2:** Rank correlations (Spearman’s rho) between fear scores on the snake, lizard, and small bird questionnaires, and EPN amplitudes (at the occipito-parietal cluster) in response to each corresponding animal category (*n* = 24).

Category	Snake	Lizard	Bird
Skin	−0.45*	−0.52**	−0.10
Partially exposed	−0.07	−0.46*	−0.23

*p < 0.05; **p < 0.01.
